# Protective enzyme activity regulation in cotton (*Gossypium hirsutum* L.) in response to *Scirpus planiculmis* stress

**DOI:** 10.3389/fpls.2022.1068419

**Published:** 2022-11-28

**Authors:** Quancheng Zhang, Jun Peng, Jungang Wang

**Affiliations:** College of Agriculture, Shihezi University, Shihezi, China

**Keywords:** chlorophyll, soluble protein, MDA, SOD, POD, CAT, defense response

## Abstract

*Scirpus planiculmis*, an important weed in rice and cotton fields, stresses crop growth and development, leading to yield loss. However, it is unclear how stressed plants respond to this weed. In this study, we analysed the stress effect of *S. planiculmis* on cotton under different weed densities, competition periods, and distribution conditions from the perspective of morphogenesis, physiological metabolism and crop yield. The effect of a low dose of herbicide on the relationship between cotton and *S. planiculmis* was also explored. The results showed that plant height, stem diameter, fresh weight, root length, boll number, single boll weight and yield of cotton all decreased with increasing *S. planiculmis* density and damage. The spatial distribution of *S. planiculmis* had no significant effect on plant height, stem diameter, fresh weight or root length of cotton, but crop yield loss decreased with increasing distance. *S. planiculmis* stress altered cotton chlorophyll, soluble protein and malondialdehyde (MDA) content, and protective enzyme activities. Compared with superoxide dismutase (SOD) and peroxidase (POD) activities, catalase (CAT) activity was increased under different *S. planiculmis* stress conditions. Therefore, we concluded that CAT plays a key role in protecting enzymes involved in defence responses. Under low-dose herbicide action, the activities of protective enzymes were increased, which helped cotton plants to resist *S. planiculmis* stress. The results revealed that regulating protective enzyme activities is important in cotton responses to *S. planiculmis* stress.

## Introduction

Cotton (*Gossypium hirsutum* L.) is an economically important crop widely planted in many parts of the world, and China is the world’s largest cotton producer (Li et al., 2021). In 2021, the cotton planting area in China reached 3.03 million hectares (National Bureau of Statistics, 2021). Weeds are one of the most important factors restricting development of the cotton industry (Wang et al., 2015). The incidence of weed damage in cotton fields can be up to 56%, causing annual yield losses of 14−16% (Li et al., 2019). Weeds are an important source of biological stress for cotton, competing for light, nutrients, water and space, and this stress occurs throughout the growth period (Vulchi et al., 2022). Weeds compete with cotton, and they cast shade, severely inhibiting growth, resulting in short cotton plants, yellow leaves, and even buds falling off, thus reducing cotton yield (Wang et al., 2015). In order avoid damage caused by weeds, it is necessary to clarify the competitive effects weeds have on cotton plants in the field.

Weed stress factors affecting cotton growth include species, density, competition period, and spatial distribution (Wang et al., 2015). Numerous studies have reported competition between weeds and cotton, examples of which include giant ragweed (*Ambrosia trifida*), goosegrass (*Eleusine indica*), redroot pigweed (*Amaranthus retroflexus*), velvetleaf (*Abutilon theophrasti*), and black nightshade (*Solanum nigrum*). (Barnett and Steckel, 2013; Ma et al., 2015a; Ma et al., 2015b; Ma et al., 2016; Zhang et al., 2022a). These studies provided insight into competition between weeds and cotton flowers at the population level. Thus, if weeds emerge earlier or at the same time as cotton seedlings, and the growth rate of the aboveground canopy of weeds exceeds that of cotton, shading will occur, and cotton will be at a competitive disadvantage, which will eventually lead to a decline in cotton yield (Ma et al., 2015b; Ma et al., 2016). Conversely, if weeds emerge later, or if they are small and adopt a creeping form, they will be shaded by cotton, and the competitiveness of the weeds is relatively weak (Ma et al., 2015a). In addition, weeds that emerge simultaneously with or earlier than cotton are more competitive than those that emerge later in the cotton growth cycle, causing greater losses in cotton yield (Barnett and Steckel, 2013). By studying the competitive effects between weeds and cotton, the critical period and density of weed control were determined, as was the control threshold (Charles et al., 2019a; Charles et al., 2019b; Charles et al., 2020; Zhang et al., 2022a). However, there are few reports on how cotton responds to weed stress from the perspective of weed and cotton physiology (Zhang et al., 2022a). Therefore, analysing the stress effects exerted by weeds on cotton from the perspective of physiological metabolism could help to further clarify the competition mechanism between weeds and cotton.

Defence responses of plants are triggered by various biotic stresses (Steinbrenner et al., 2022). In these complex stress response systems, basic metabolism, specialized metabolism and protective enzymes are known to play important roles (Ling et al., 2022; Swinnen et al., 2022). For example, in the face of attack by most herbivorous insects, plant protective enzyme activities and basal metabolic capacity are enhanced, effectively improving tolerance and reducing damage by herbivorous insects (Castano-Duque et al., 2018; Gao et al., 2022). Although weeds cannot induce defence responses of plants through direct damage, they can stimulate defence systems through allelopathy (Li et al., 2022a). Allelopathy affects plant cell structure, photosynthesis, respiration, hormone content, receptor enzyme activity and gene expression by releasing chemicals (Kato-Noguchi et al., 2017; Qin et al., 2018; Siyar et al., 2019; Mardani et al., 2019). Allelopathic plants reduce interference of allelopathy by regulating related tissue structure, basal metabolism, secondary metabolism, protective enzyme activity and other defensive measures (Šoln et al., 2022). Especially in plant communities with population competition, plants reduce the adverse effects of allelopathy on competition through defensive responses (Uesugi et al., 2019; Chia and Bittencourt-Oliveira, 2021). Competition between weeds and plants is obviously related to this process. However, when studying competition between weeds and plants, the competition mechanism from the perspective of plant defence metabolism is not usually investigated, even though it could be useful.

*Scirpus planiculmis* Fr. Schmidt is a perennial herb of the family Cyperaceae. In China, Korea, Japan, and other countries and regions, *S. planiculmis* is a common malignant weed in rice fields (Lee et al., 1991; Choi et al., 2000a). In Europe, it is considered to be a potential plant for widespread expansion (Hroudová et al., 2014). Previous studies have determined the economic threshold of *S. planiculmis* in rice fields (Kwon et al., 2008; Kwon et al., 2011) and screened chemical control agents (Choi et al., 2000b; Hwang et al., 2006; Park et al., 2011). However, with changes in cropping systems and cultivation patterns, *S. planiculmis* has become a major weed in cotton fields in Xinjiang and the Yellow River basin in China (Fang et al., 2016; Huang et al., 2020), leading to cotton yield reductions of 1.86−61.50% (Cai et al., 2020a). Due to the diversification of the breeding methods for *S. planiculmis*, plants can be sexually propagated through seeds and asexually propagated through bulbs (Yang et al., 2021; Zhang et al., 2022b). Strong saline-alkaline tolerance (Hroudová et al., 2014; An et al., 2021) and plastic film mulching in cotton fields provides a suitable growth environment (Hroudová et al., 2007; Liu et al., 2016), resulting in a density of up to 1.5×10^6^ *S. planiculmis* plants per hm^2^ in cotton fields in Xinjiang, which severely inhibits nutrient uptake and growth of cotton (Cai et al., 2020a; Cai et al., 2021).

Compared with C_3_ plants (cotton), C_4_ plants (*S. planiculmis*) are better at absorbing CO_2_, water, nutrients, and light (Li and Yang, 2020). Therefore, during competition between the two, cotton is weaker than *S. planiculmis*. Our previous results showed that plant height, stem diameter, number of nodes on the main stem, boll number, and fiber quality of cotton were reduced with continuous extension of damage time and increase in the density of *S. planiculmis*, which eventually led to a decrease in cotton yield (Cai et al., 2020b; Cai et al., 2021). During the cotton flowering and boll-forming stage, *S. planiculmis* reduced potassium and soluble sugar content in cotton leaves under different population densities, and inhibited the accumulation of nutrients in cotton (Cai et al., 2020a). Further studies showed that the rhizosphere exudates of *S. planiculmis* reduced the content of cytokinin and auxin in cotton leaves, and significantly inhibited the growth of underground parts of cotton, including main root length, lateral root length and fresh weight (Cai, 2020c). However, how cotton responds to *S. planiculmis* stress through physiological metabolism has not been reported.

In this study, we conducted a 2-year experiment in 2019−2020, and these were field experiment. (1) Different densities of *S. planiculmis* were established, and the effects on the growth and physiological metabolism of cotton were measured at different stages (seedling, budding, flowering and boll); (2) the competition period of *S. planiculmis* and cotton was controlled to determine the effects of *S. planiculmis* on the growth and physiological metabolism of cotton; (3) by changing the distribution distance between *S. planiculmis* and cotton, effects on the growth and physiological metabolism of *S. planiculmis* on cotton were determined; (4) the physiological metabolic responses of cotton to *S. planiculmis* were further explored under herbicide mediation. The results illuminate the physiological response mechanism of cotton to *S. planiculmis* from the perspective of plant defences.

## Materials and methods

### Experimental field and plants

The experiment was carried out at the Shihezi University Educational Test Site, Xinjiang, China (44°C22′ N, 86°C05′ E) from 2019 to 2020. The soil was loam, pH 7.8, containing 7.22 mg kg^-1^ organic carbon, 1.43 g kg^-1^ total nitrogen, 52.58 mg kg^-1^ alkaline hydrolysis nitrogen, 166.33 mg kg^-1^ available potassium and 7.27 mg kg^-1^ available phosphorus. The soil porosity was 45.17%, and the field capacity was 35.02%. The land had been growing cotton for more than 5 years, and the base number of *S. planiculmis* was large, with a density range of 50−80 plants/m^2^.

The cotton variety used was Xinluzao 60 (upland cotton, provided by the Institute of Cotton Research, Chinese Academy of Agricultural Sciences, Zhengzhou, China), and seeds were treated with imidacloprid seed coating agent (Gaucho Bayer Crop Science; 600 mL/100 kg seeds). Seeds were sown on 21 April, 2019 and 25 April, 2020. Seeding was carried out under a mulch film at a width of 1.5 m. Rows were spaced at (20 + 60 + 20) cm, cotton plants were spaced ≥9 cm, and the planting density was 150,000 plants/hm^2^.

### Experimental field management

Prior to sowing, the experimental field was covered with a plastic film, and two drip irrigation lines (Beijing Luckrain Inc., Beijing, China) were installed under each plastic film. The drip irrigation line had an inner diameter of 2.5 cm, an emitter distance of 50 cm, a flow rate of 2.7 L h^-1^, and 4500 kg hm^-2^ of oil residue (13% N, 2% P_2_O_5_ and 16% K_2_O) was applied as base fertiliser. In addition, 75 kg hm^-2^ of urea (46% N) and 216 kg hm^-2^ of triple superphosphate (45% P_2_O_5_) were applied throughout the growth period. Drip irrigation under the film was applied eight times during the whole cotton growth period. The irrigation time in 2020 was 53, 65, 76, 87, 97, 108, 119 and 127 days after cotton emergence, respectively. In 2021, it was 58, 67, 78, 89, 99, 110, 117 and 125 days after cotton emergence, respectively. Additionally, 98% Mepiquat chloride (MC; Sichuan Guoguang Agrochemical Co., Ltd.,Chengdu, China) was applied to control vegetative growth. MC solution at 208 g hm^-2^ concentration was sprayed five times throughout the whole cotton growth period. A 6 g hm^-2^ MC solution was sprayed from the cotyledon stage to the two-leaf stage, and 11 g hm^-2^ was sprayed at the 5- to 7-leaf stage. Moreover, 26, 45 and 120 g hm^-2^ MC was sprayed 2 days before the first irrigation, 2 days before the second irrigation, and 5−7 days after topping. To hasten crop maturation and defoliation, 600 g hm^-2^ 50% thidiazuron defoliant (Sichuan Guoguang Agrochemical Co., Ltd.) combined with 3000 mL hm^-2^ 40% ethephon (Sichuan Guoguang Agrochemical Co., Ltd.) was used in both years. Pest and disease control were carried out according to standard management practices, mainly to control cotton aphids, thrips and spider mites. Weeds were manually removed every 30 days retaining only experimental *S. planiculmis*.

### Chemicals

Trifloxysulfuron (75%) (Swiss Syngenta Crop Protection Co., Ltd., Switzerland) and prometryn (40%) (Zhejiang Changxing First Chemical Co., Ltd., Changxing, China) were applied as wettable powders.

### Effects of *S. planiculmis* density on growth of *S. planiculmis* and cotton

Based on the occurrence of *S. planiculmis* in cotton fields and preliminary test results (Cai et al., 2020a; Cai et al., 2021), we set *S. planiculmis* densities of 0, 20, 40, 60, 80 and 100 plants/m^2^, totalling six treatments. The experiment used a completely randomized block design with an area of 2.3 m^2^ (2.3 m × 1 m) per plot. The weed densities established for a whole plot (Swanton et al., 2015). Each treatment was repeated five times, totalling 30 plots. After cotton emergence, the emergence rate of *S. planiculmis* in each plot was investigated. If the density of *S. planiculmis* was higher than the set density, excess *S. planiculmis* plants were removed. If the density of *S. planiculmis* was lower than the set density, *S. planiculmis* plants were transplanted to the set density. During the whole growth period of cotton, other weeds in the plot were artificially removed leaving only *S. planiculmis*.

Plant height, stem diameter, fresh weight and root length of *S. planiculmis* in each plot were measured at the squaring stage of cotton, and leaves of *S. planiculmis* were collected. Levels of chlorophyll, soluble protein, malondialdehyde (MDA) and the activities of SOD, POD and CAT enzymes were determined in the laboratory. Six *S. planiculmis* plants were measured from each plot, and 30 *S. planiculmis* plants were measured for each treatment. Plant height, stem diameter, fresh weight and root length of cotton in each plot were measured at the seedling, budding, flowering and boll stages, and cotton leaves were collected. Levels of chlorophyll, soluble protein, MDA, and the activities of SOD, POD and CAT enzymes were measured in the laboratory. Six cotton plants were measured from each plot, and 30 cotton plants were measured for each treatment. At harvest, the number of cotton bolls, single boll weight, and yield were measured for each treatment. All remaining cotton plats in each plot were measured.

### Effects of planting time of *S. planiculmis* on the growth of *S. planiculmis* and cotton

In the experiment, *S. planiculmis* seedlings were planted in April (April 21, 2019 and April 22, 2020), May (May 21, 2019 and May 2020), and June (June 21, 2019 and June 22, 2020). The planting density was set at 60 plants/m^2^ (Cai et al., 2020b). Based on the frequency of weeding in cotton fields and the results of preliminary experiments (Cai et al., 2020a), we set three competition periods for *S. planiculmis* and cotton: 0 (control, no *S. planiculmis*), 30 and 60 days (Swanton et al., 2015). Therefore, there were nine treatments in the experiment, and each treatment was repeated five times, totalling 45 plots, and each plot area was 2.3 m^2^ (2.3 m × 1 m). During the experiment, seedlings of *S. planiculmis* that died within 1 week were replanted, the density of *S. planiculmis* was kept at the set value, and weeds remaining in the plot were removed. Plant height, stem diameter, fresh weight and root length under different *S. planiculmis* planting times were measured on June 30, and leaves were collected to determine the contents of chlorophyll, soluble protein, MDA and the activities of SOD, POD and CAT enzymes. Six *S. planiculmis* plants were measured per plot, and 30 *S. planiculmis* plants were collected for each treatment. Plant height, stem diameter, fresh weight and root length of cotton were measured at 0, 30 and 60 days after planting. The contents of chlorophyll, soluble protein, MDA and the activities of SOD, POD and CAT enzymes were determined. Six cotton plants were measured from each plot, and 30 cotton plants were measured for each treatment. At harvest, the number of cotton bolls, single boll weight, and the cotton yield under different *S. planiculmis* planting times were measured.

### Effects of *S. planiculmis* spatial distribution on the growth of *S. planiculmis* and cotton

After cotton emergence, the distance between *S. planiculmis* and cotton was set to 0, 5, 10, 15 and 20 cm, totalling five treatments. Each treatment was repeated five times, with 25 plots covering 2.3 m^2^ (2.3 m × 1 m) per plot. The number of *S. planiculmis* planted at all distances was eight (with cotton at the centre, a *S. planiculmis* plant was planted at each of the eight compass points (east, west, south, north, northeast, southeast, northwest and southwest) at different distances (Swanton et al., 2015). During the experiment, seedlings of *S. planiculmis* without emergence and death within 1 week were replanted in a timely manner. At the 5-leaf stage of *S. planiculmis*, plant height, stem diameter, fresh weight and root length of *S. planiculmis* and cotton were measured, and leaves were collected to determine chlorophyll, soluble protein, MDA content and SOD, POD and CAT enzyme activities. Six *S. planiculmis* and six cotton plants were measured from each plot, and 30 *S. planiculmis* and 30 cotton plants were included for each treatment. At harvest, the number of cotton bolls, single boll weight, and cotton yield were measured.

### Effects of spraying herbicides on the physiological metabolism of cotton and *S. planiculmis*

According to the results of previous pesticide screening (Peng et al., 2012), at the 5-leaf stage of *S. planiculmis*, herbicide (0.015 g L^-1^ 75% trifloxysulfuron + 4 g L^-1^ 40% prometryn) was evenly sprayed using a 3WBD-20 L knapsack electric sprayer at a water amount of 500 kg hm^-2^. After 5 days, the contents of chlorophyll, soluble protein, MDA and the activities of SOD, CAT and POD enzymes in leaves of cotton and *S. planiculmis* were determined. Six *S. planiculmis* and six cotton plants were measured for each plot, and 30 *S. planiculmis* and 20 cotton plants were included for each treatment.

### Analysis of plant height, stem diameter, root length and fresh weight of cotton and *S. planiculmis*

Twenty cotton plants were randomly selected from each treatment for analysis of plant height, stem diameter, root length and fresh weight. Plant height was determined as the length from the stem base to the growing point. Stem diameter was measured with a Vernier calliper. Root length was the length of the main root. Fresh weight was measured with an electronic balance.

### Analysis of chlorophyll, soluble protein and MDA levels and protective enzyme activities in cotton and *S. planiculmis*

Chlorophyll content was measured using the acetone ethanol method (Arnon, 1949). Soluble protein was determined using the G-250 dye colorimetric method (Bradford, 1976). The MDA content was determined based on the thiobarbituric acid method (Dhindsa et al., 1981). CAT, POD and SOD activities were determined using the guaiacol method (Chance and Maehly, 1955), the UV absorption method (Amalo et al., 1994) and the riboflavin-NBT method (Giannopolities and Ries, 1977), respectively.

### Analysis of boll number, single boll weight and yield of cotton

Seed cotton from each plot was hand-picked on 15 October, sun-dried, and weighed. One hundred fully opened bolls were sampled to calculate individual boll weight and lint percentage. Boll number was determined by counting bolls (> 2 cm in diameter) for each plant on 19 September and 21 September in 2019 and 2020, respectively.

### Statistical analysis

Microsoft Excel 2012 was used for data processing and plotting. SPSS 20.0 was used for all statistical analyses, yielded mean values and standard errors. The LSD method in one-way ANOVA and t-test were used to test for significance between differences in means.

## Results

### Effects of population density of *S. planiculmis* on growth and physiological indices of *S. planiculmis*

**Figure 1:**
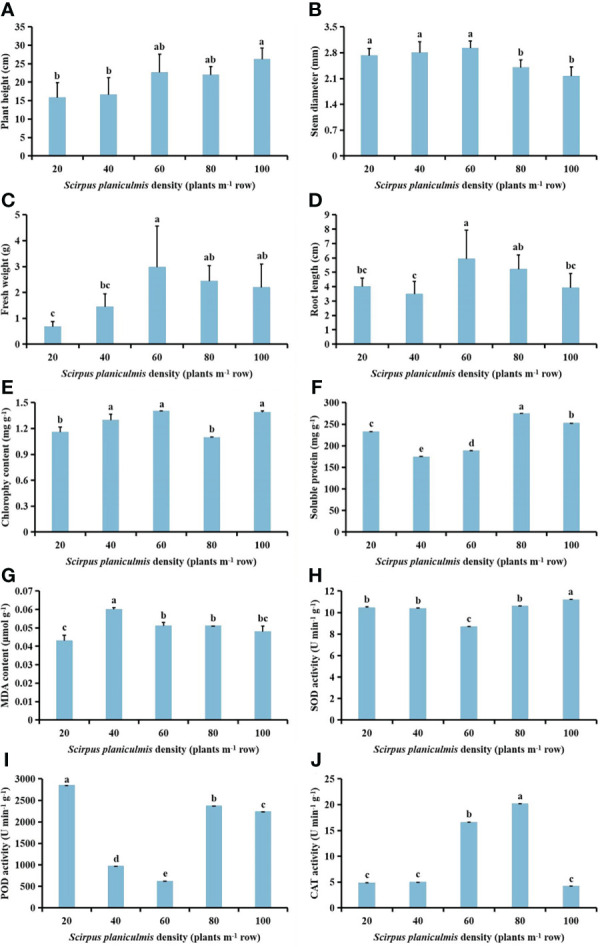
Effects of different *Scirpus planiculmis* densities on **(A)** plant height, **(B)** stem diameter, **(C)** fresh weight, **(D)** root length, **(E)** chlorophyll, **(F)** soluble protein **(G)** and malondialdehyde (MDA) content, and **(H)** superoxide dismutase (SOD), **(I)** peroxidase (POD) and **(J)** catalase (CAT) activities in *S. planiculmis*. The mean value ± standard data were presented in the figure. Different alphabetical letters indicate the significant differences between different *S. planiculmis* density (p <0.05).

With increasing *S. planiculmis* density, plant height tended to increase, and stem diameter tended to decrease; root length, fresh weight, MDA content and CAT activity first increased then decreased; chlorophyll content showed the opposite trend to soluble protein content and POD activity, while SOD activity first decreased then increased in *S. planiculmis* (**Figure 1**). When the density was 100 plants m^-2^, plant height was the highest (26.24 cm) and stem diameter was the lowest (2.16 mm; **Figure 1A, B**). When the density was 60 plants m^-2^, the fresh weight and root length of *S. planiculmis* reached maximum values of 2.97 g and 5.94 cm, respectively (**Figure 1C, D**). At this time, the chlorophyll content was also highest (1.40 mg g^-1^), 21.91% higher than that of 80 plants m^-2^ (*p <*0.05; **Figure 1E**). The highest soluble protein content was 274.53 mg g^-1^ when the density was 80 plants m^-2^ (**Figure 1F**). The MDA content was lowest at a density of 20 plants m^-2^, and highest at 40 plants m^-2^, at 0.043 and 0.06 μmol g^-1^, respectively (**Figure 1G**). When the density was 60 plants m^-2^, SOD and POD activities of *S. planiculmis* were lowest, at 8.67, and 616.73 U min^−1^ g^−1^, respectively (**Figure 1H, I**). When the density of *S. planiculmis* was 80 plants m^-2^, CAT activity was highest (20.18 U min^−1^ g^−1^), 18.11% higher than that of 60 plants m^-2^ (*p <*0.05; **Figure 1J**).

### Effects of *S. planiculmis* population density on agronomic traits and physiological indices in cotton

**Figure 2:**
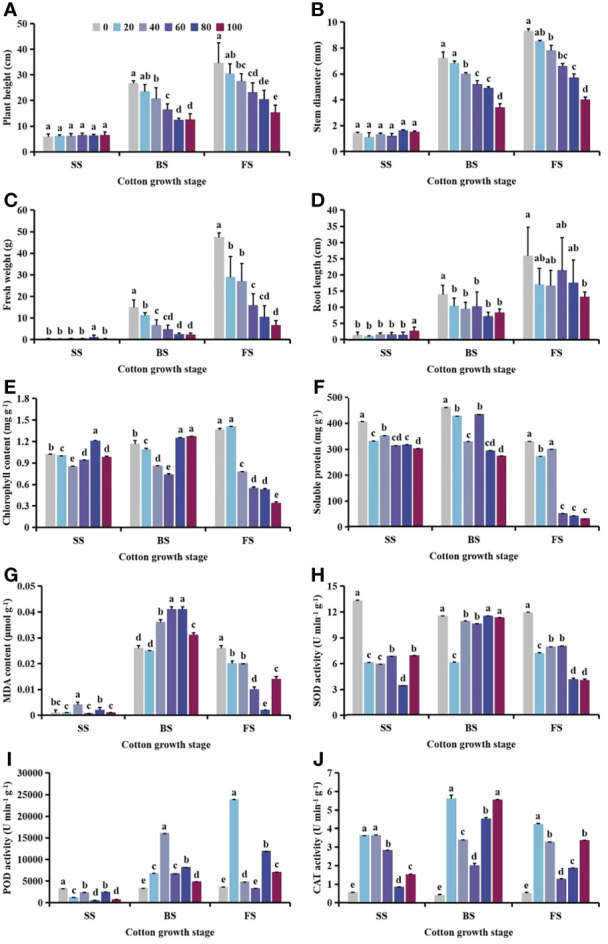
Under seedling, budding, and flowering and boll stages of cotton, the **(A)** plant height, **(B)** stem diameter, **(C)** fresh weight, **(D)** root length, **(E)** chlorophyll, **(F)** soluble protein **(G)** and malondialdehyde (MDA) content, and **(H)** superoxide dismutase (SOD). **(I)** peroxidase (POD) and **(J)** catalase (CAT) activities of cotton at different *Scirpus planiculmis* densities. SS, seedling stage, BS, budding stage, FS, flowering and boll stage. Results are mean ± standard error (*p* <0.05). The mean value ± standard data were presented in the figure. Different alphabetical letters indicate the significant differences between different *S. planiculmis* density (*p* <0.05).

With increasing *S. planiculmis* density, plant height, stem diameter, fresh weight and root length of cotton at the seedling stage (SS) were barely affected, while plant height, stem diameter, fresh weight and root length at the budding stage (BS), and flowering and boll stage (FS) showed a decreasing trend (**Figure 2A-D**). When the densities of *S. planiculmis* were 20, 40 and 60 plants m^-2^, the chlorophyll content of cotton at the seedling, budding, flowering and boll stages was the lowest, 17.24%, 36.51% and 75.35% lower than that of controls (0 plants m^-2^), respectively (*p <*0.05; **Figure 2E**). The soluble protein content of cotton at each stage reached the lowest value at a *S. planiculmis* density of 100 plants m^-2^, 25.13%, 40.50% and 90.94% lower than that of controls (0 plants m^-2^), respectively (*p <*0.05; **Figure 2F**). At the flowering and boll stage, the MDA content under all densities was significantly lower than that of controls (0 plants m^-2^), and when the density of *S. planiculmis* was 80 plants m^-2^, the MDA content was lowest, 92.31% lower than that of controls (*p <*0.05, **Figure 2G**). At each growth stage, the SOD activity of cotton under all densities of *S. planiculmis* was lower than that of controls (0 plants m^-2^), while CAT activity was higher than that of controls (**Figure 2H, J**). POD activity at the seedling stage was significantly lower than that of controls (0 plants m^-2^), but at the budding, flowering and boll stages POD activity was increased significantly, reaching a maximum at 40 and 20 plants m^-2^, respectively, increased by 78.96% and 84.97% compared with controls (**Figure 2I**).

### Effects of growth stage on agronomic traits and physiological indices in cotton under different *S. planiculmis* population density

**Figure 3:**
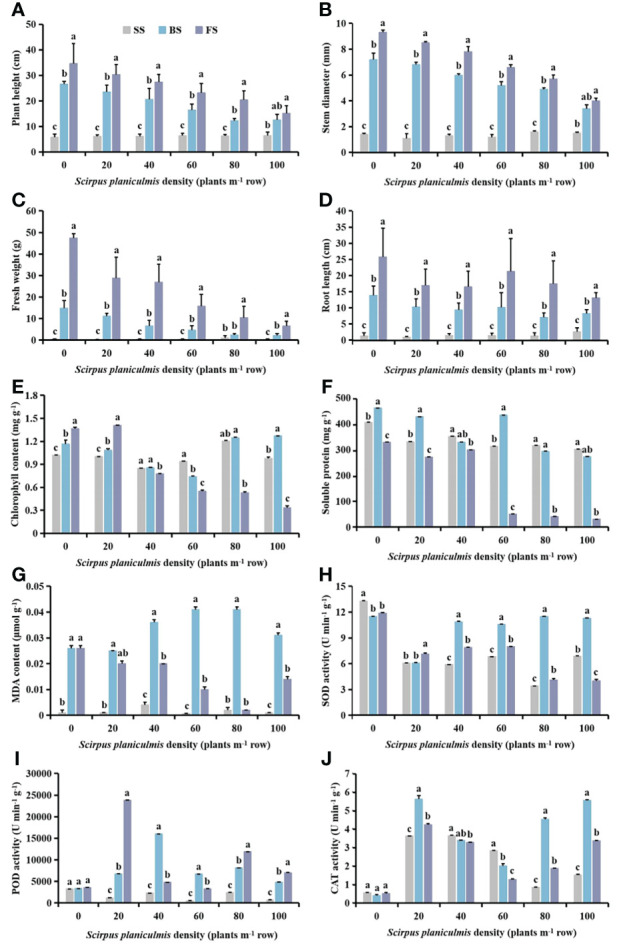
Under different *Scirpus planiculmis* densities, the **(A)** plant height, **(B)** stem diameter, **(C)** fresh weight, **(D)** root length, **(E)** chlorophyll, **(F)** soluble protein and **(G)** malondialdehyde (MDA) content, and **(H)** superoxide dismutase (SOD). **(I)** peroxidase (POD) and **(J)** catalase (CAT) activities of cotton at seedling, budding, and flowering and boll stages, SS, seedling stage; BS, budding stage, FS, flowering and boll stage. The mean value ± standard data were presented in the figure. Different alphabetical letters indicate the significant differences between different cotton growth stage (*p* <0.05).

Plant height, stem diameter, fresh weight and root length of cotton increased with the growth of cotton under different densities of *S. planiculmis* (**Figures 3A-D**). When the density of *S. planiculmis* was ≤20 plants m^-2^, the chlorophyll content of cotton increased with the growth of cotton; when the density of *S. planiculmis* was 40−60 plants m^-2^, the chlorophyll content of cotton decreased with the growth of cotton; when the density of *S. planiculmis* was ≥80 plants m^-2^, the chlorophyll content of the budding stage was higher than that of the other stages, especially when the density of *S. planiculmis* was 100 plants m^-2^; the chlorophyll content of the budding stage was 23.05% and 73.56% higher than that of the seedling stage and the flowering and boll stage (*p <*0.05; **Figure 3E**). When the densities of *S. planiculmis* were 0, 20 and 60 plants m^-2^, the soluble protein content in the budding stage was significantly higher than in other stages, while the soluble protein content in cotton decreased with the growth of cotton at densities of 40, 80 and 100 plants m^-2^ (**Figure 3F**). At different densities of *S. planiculmis*, the MDA content in cotton at the seedling stage was lowest, and that in the budding stage was highest (**Figure 3G**). When the density of *S. planiculmis* was ≥40 plants m^-2^, SOD activity in cotton at the budding stage was significantly higher than that in other stages (**Figure 3H**). When the density of *S. planiculmis* was 0 plants m^-2^, there was no significant change in POD and CAT activities of cotton at different stages. When the density was 20, 80 and 100 plants m^-2^, POD activity was highest at the flowering and boll stage, and CAT activity was highest at the budding stage (**Figures 3I, J**).

### Effects of *S. planiculmis* population density on yield in cotton

**Figure 4:**
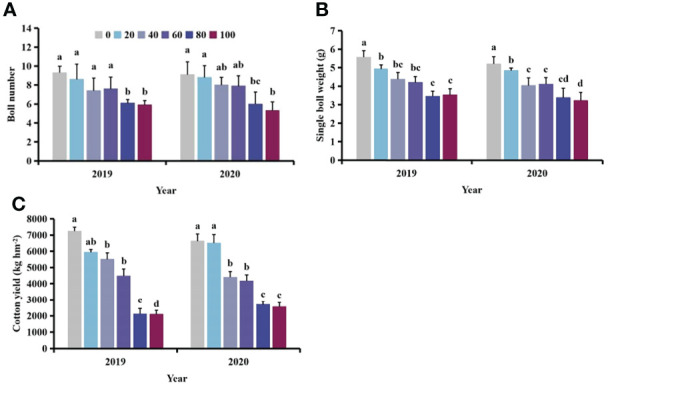
Effects of different *Scirpus planiculmis* densities on cotton **(A)** boll number, **(B)** single boll weight and **(C)** cotton yield. The mean value ± standard data were presented in the figure. Different alphabetical letters indicate the significant differences between different *S. planiculmis* density (*p* <0.05).

With increasing density of *S. planiculmis*, the boll number, boll weight and yield of cotton showed a downward trend (**Figure 4**). When the density of *S. planiculmis* was 100 plants m^-2^, the number of cotton bolls was lowest, 36.56−41.76% lower than that of controls (0 plants/m^2^; **Figure 4A**). Compared with controls, single boll weight decreased by 37.85−38.07% and cotton yield decreased by 61.50−71.22% (**Figures 4B, C**).

### Effects of planting time of *S. planiculmis* on growth and physiological indices of *S. planiculmis*

**Figure 5:**
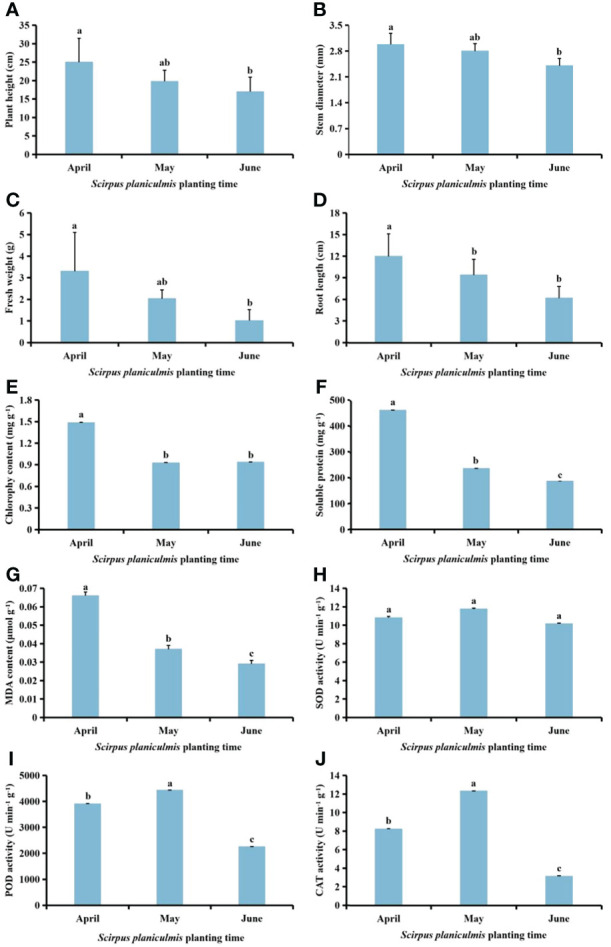
Effects of *Scirpus planiculmis* planting time on **(A)** plant height, **(B)** stem diameter, **(C)** fresh weight, **(D)** root length, **(E)** chlorophyll, **(F)** soluble protein and **(G)** malondialdehyde (MDA) content, and **(H)** superoxide dismutase (SOD), **(I)** peroxidase (POD) and **(J)** catalase (CAT) activities in *S. planiculmis*. The mean value ± standard data were presented in the figure. Different alphabetical letters indicate the significant differences between different *S. planiculmis* planting time (*p* <0.05).

With delayed planting time, plant height, stem diameter, fresh weight, root length, and chlorophyll, soluble protein and MDA contents of *S. planiculmis* in June were significantly lower than in April (*p <*0.05), while the activities of SOD, POD and CAT in May were higher than in April and June (**Figure 5**). Compared with *S. planiculmis* planted in April, plant height, stem diameter, fresh weight and root length of *S. planiculmis* planted in June were decreased by 32.11%, 19.46%, 69.09% and 48.33%, respectively (*p <*0.05; **Figures 5A-D**), while chlorophyll, soluble protein and MDA contents were decreased by 36.84%, 59.87% and 56.06%, respectively (*p <*0.05, **Figures 5E-G**). The activities of SOD, POD and CAT of *S. planiculmis* planted in June were decreased by 13.58%, 49.21% and 74.54%, respectively, compared with those planted in May (**Figures 5H-J**).

### Effects of competition periods of *S. planiculmis* on agronomic traits and physiological indices in cotton

**Figure 6:**
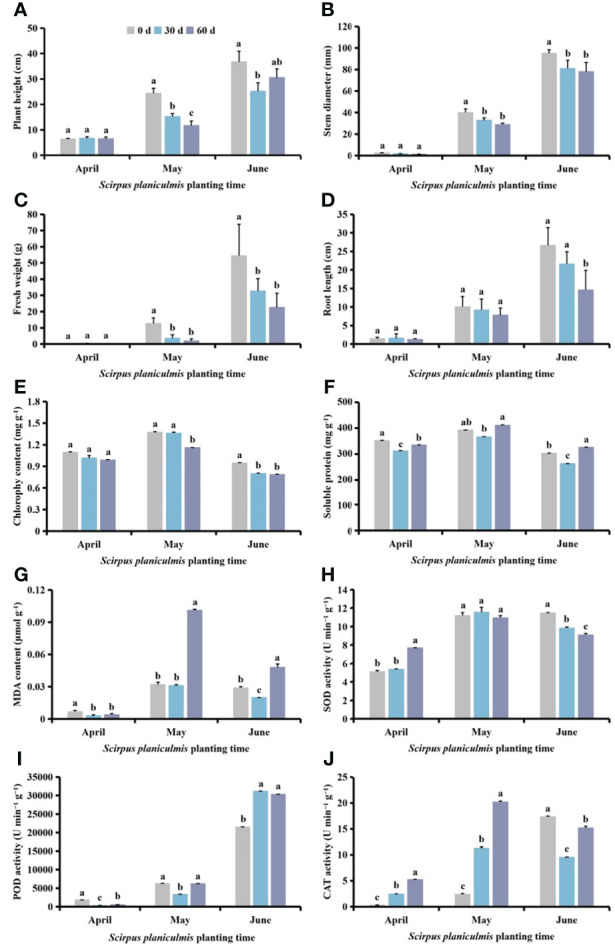
Under different *Scirpus planiculmis* planting time, the **(A)** plant height, **(B)** stem diameter, **(C)** fresh weight, **(D)** root length, **(E)** chlorophyll, **(F)** soluble protein and **(G)** malondialdehyde (MDA) content, and **(H)** superoxide dismutase (SOD), **(I)** peroxidase (POD) and **(J)** catalase (CAT) activities in cotton at different competition periods The mean value ± standard data were presented in the figure. Different alphabetical letters indicate the significant differences between different competition periods (*p* <0.05).

Among different planting times of *S. planiculmis*, planting in April had no significant effect on plant height, stem diameter, fresh weight, root length or chlorophyll content (*p >*0.05; **Figures 6A-E**). Compared with controls (0 days), soluble protein and MDA contents and POD activity of cotton were significantly decreased after competition with *S. planiculmis* for 60 and 30 days (*p <*0.05), while soluble protein and MDA contents and POD activity of cotton were lowest at 30 days, at 312.20 mg g^-1^, 0.003 μmol g^−1^ and 350.25 U min^−1^ g^−1^, respectively (**Figures 6F, G, I**). SOD and CAT activities were significantly increased, and they were highest at 60 days, at 7.67 and 5.29 U min^−1^ g^−1^, respectively (**Figure 6H, J**). In May, plant height, stem diameter, fresh weight, root length and chlorophyll content of cotton decreased with increasing competition duration, compared with controls (0 days), and plant height, stem diameter, fresh weight, root length and chlorophyll content of cotton were decreased by 52.05%, 27.50%, 84.34%, 22.62% and 9.6%, respectively (*p <*0.05; **Figures 6A-E**). The soluble protein and MDA contents and POD activity of cotton were lowest at 30 days, at 366.20 mg g^-1^, 0.031 μmol g^−1^ and 3366.68 U min^−1^ g^−1^, respectively (**Figures 6F, G, I**). There were no significant differences in SOD activity among different treatments (*p >*0.05), but CAT activity increased with extended competition duration, by 88.08% and 78.62%, respectively, at 30 and 60 days (*p <*0.05; **Figures 6H, J**). In June, compared with controls (0 days), plant height, stem diameter, fresh weight, root length and chlorophyll content of cotton seedlings following competition with *S. planiculmis* for 30 and 60 days were decreased (**Figures 6A-E**). Soluble protein and MDA contents and CAT activity were lowest at 30 days, 13.29%, 31.03%, and 41.57% lower than those of controls (0 days), respectively (**Figures 6F, G, J**). SOD activity was decreased by 14.37% and 20.71% (*p <*0.05), and POD activity was increased by 30.96% and 29.10% at 30 and 60 days, respectively (*p <*0.05; **Figures 6H, I**).

### Effects of planting time of *S. planiculmis* on agronomic traits and physiological indices in cotton

**Figure 7:**
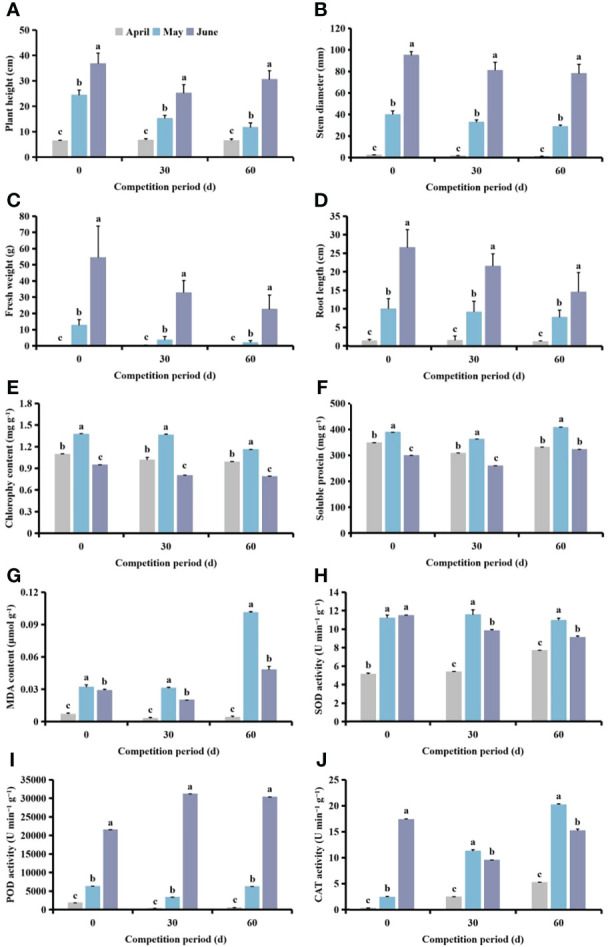
Under different competition periods, the **(A)** plant height, **(B)** stem diameter, **(C)** fresh weight, **(D)** root length, **(E)** chlorophyll, **(F)** soluble protein and **(G)** malondialdehyde (MDA) content, and **(H)** superoxide dismutase (SOD) **(I)** peroxidase (POD) and **(J)** catalase (CAT) activities in cotton at different *Scirpus planiculmis* planting time. The mean value ± standard data were presented in the figure. Different alphabetical letters indicate the significant differences between different different *Scirpus planiculmis* planting time (*p* <0.05).

Following different competition periods, plant height, stem diameter, fresh weight and root length of cotton increased with delayed *S. planiculmis* planting time. Plant height, stem diameter, fresh weight, root length and chlorophyll content of cotton planted in April and May were significantly lower than for those planted in June (*p <*0.05, **Figures 7A-D**). The chlorophyll, soluble protein and MDA contents of cotton in May were significantly higher than those in April and June (*p <*0.05; **Figures 7E-G**). The activities of SOD, POD and CAT in cotton were lowest in April, significantly lower than those in May and June (*p <*0.05; **Figures 7H-J**).

### Effects of competition periods of *S. planiculmis* on yield in cotton

**Figure 8:**
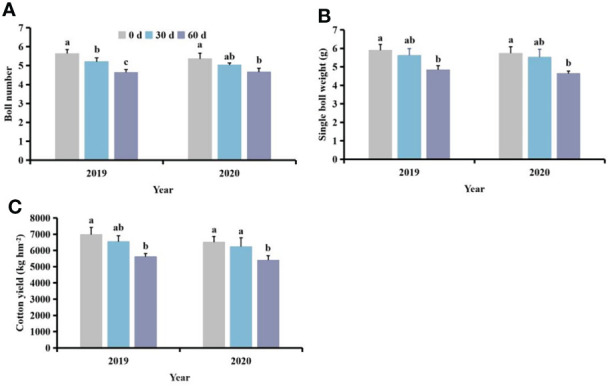
Effects of different competition periods on cotton **(A)** boll number, **(B)** single boll weight, and **(C)** cotton yield. The mean value ± standard data were presented in the figure. Different alphabetical letters indicate the significant differences between different competition periods (*p* <0.05).

Following different competition periods with *S. planiculmis*, the number of cotton bolls, single boll weight and yield of cotton following competition for 60 days were lowest, 13.06−17.73%, 17.89−19.12% and 17.02−19.50% lower than those of controls (0 days), respectively (*p <*0.05; **Figure 8**).

### Effects of spatial distribution of *S. planiculmis* on agronomic traits and physiological indices in cotton and *S. planiculmis*

**Figure 9:**
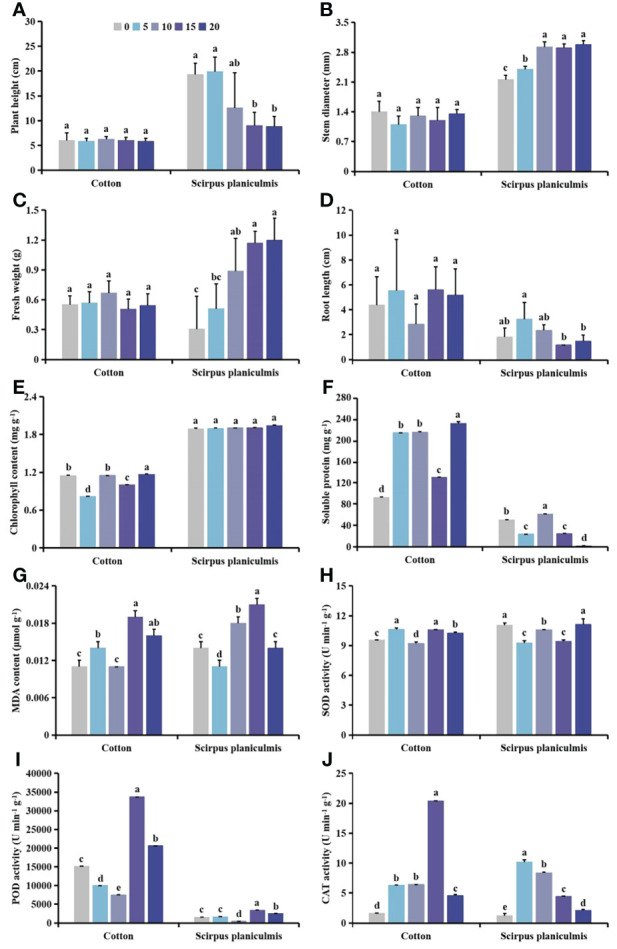
The **(A)** plant height, **(B)** stem diameter, **(C)** fresh weight, **(D)** root length, **(E)** chlorophyll, **(F)** soluble protein and **(G)** malondialdehyde (MDA) content, and **(H)** superoxide dismutase (SOD), **(I)** peroxidase (POD) and **(J)** catalase (CAT) activities in cotton and *S. planiculmis* with different spatial distribution of *Scirpus planiculmis*. The mean value ± standard data were presented in the figure. Different alphabetical letters indicate the significant differences between different distances (*p* <0.05).

The distance between cotton and *S. planiculmis* plants had no significant effect on plant height, stem diameter, fresh weight and root length of cotton, but with increasing distance, plant height and root length of *S. planiculmis* decreased, and stem diameter and fresh weight increased (**Figures 9A-D**). There was no significant difference in chlorophyll content of *S. planiculmis* at different distances (*p >*0.05), but when the distance was 5 cm, the chlorophyll content of cotton was lowest, 28.50% lower than that at 0 cm (*p <*0.05, **Figure 9E**). Compared with a distance of 0 cm, the soluble protein content of cotton at 5−20 cm distance was significantly increased, and that at 15 cm was 30.28% higher than that at 0 cm (*p <*0.05). When the distance was 20 cm, the soluble protein content of *S. planiculmis* was lowest, 76.85% lower than that at 0 cm (*p <*0.05; **Figure 9F**). When the distance between *S. planiculmis* and cotton was 15 cm, the MDA content of *S. planiculmis* and cotton was highest, 0.019 and 0.021 μmol g^−1^, respectively (**Figure 9G**). With differences in distance, cotton and *S. planiculmis* showed opposite trends (**Figure 9H**). When the distance was 15−20 cm, POD activity of cotton and *S. planiculmis* was significantly higher than that at 0 cm (*p <*0.05), and POD activity of cotton and *S. planiculmis* was lowest at 10 cm, at 7484.42, and 466.23 μmol/g/min, respectively (**Figure 9I**). Compared with 0 cm, CAT activity of cotton and *S. planiculmis* at a distance of 5−20 cm was increased significantly (*p <*0.05). When the distance was 15 cm, CAT activity of cotton was highest, 92.03% higher than that at 0 cm (*p <*0.05). When the distance was 5 cm, CAT activity of *S. planiculmis* was highest, 87.80% higher than that at 0 cm (*p <*0.05; **Figure 9J**).

**Figure 10:**
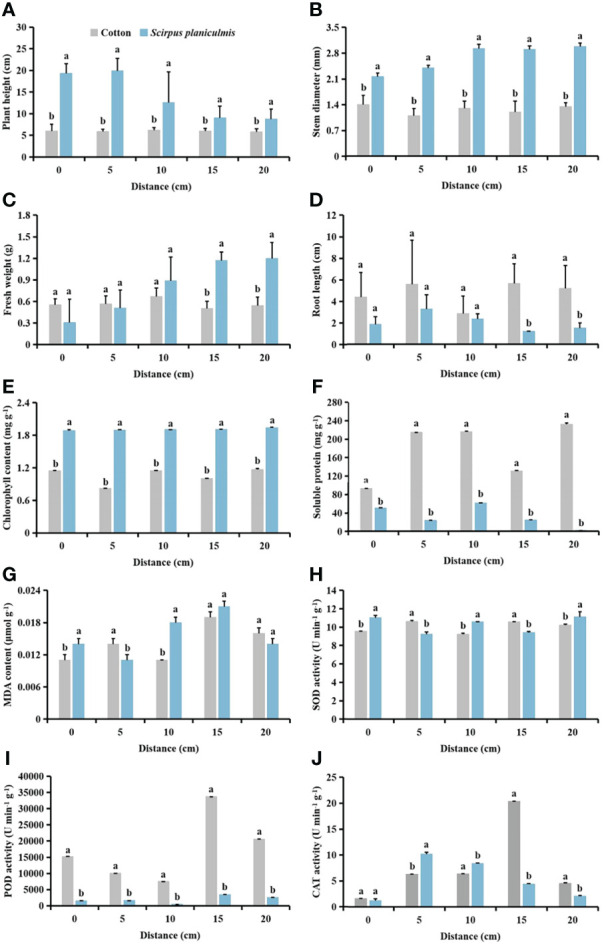
Under different spatial distribution of *Scirpus planiculmis*, the **(A)** plant height, **(B)** stem diameter, **(C)** fresh weight, **(D)** root length, **(E)** chlorophyll, **(F)** soluble protein and **(G)** malondialdehyde (MDA) content, and **(H)** superoxide dismutase (SOD). **(I)** peroxidase (POD) and **(J)** catalase (CAT) activities in cotton and *S. planiculmis*. The mean value ± standard data were presented in the figure. Different alphabetical letters indicate the significant differences between cotton and *S. planiculmis* (*p* <0.05).

At different distances, plant height and stem diameter of *S. planiculmis* were significantly smaller than those of cotton (**Figures 10A, B**). When the distance was ≥15 cm, fresh weight of cotton was significantly lower than that of *S. planiculmis* (*p <*0.05), while root length of cotton was significantly higher than that of *S. planiculmis* (*p <*0.05; **Figures 10C, D**). With increasing distance between *S. planiculmis* and cotton, the chlorophyll content of *S. planiculmis* was significantly higher than that of cotton, while the soluble protein content of cotton was significantly higher than that of *S. planiculmis* (*p <*0.05; **Figures 10E, F**). When the distance was >15 cm, there was no significant difference in MDA content between *S. planiculmis* and cotton (*p >*0.05; **Figure 10G**). When the distance was 0, 10 and 20 cm, SOD activity of *S. planiculmis* was higher than that of cotton. When the distance was 5 and 15 cm, SOD activity of *S. planiculmis* was lower than that of cotton (*p <*0.05; **Figure 10H**). POD activity of cotton was higher than that of *S. planiculmis* at each distance (*p <*0.05; **Figure 10I**). When the distance was 0 cm, there was no significant difference in CAT activity between *S. planiculmis* and cotton (*p >*0.05), while at 5−10 cm CAT activity of *S. planiculmis* was higher than that of cotton (*p <*0.05), and at 15−20 cm CAT activity of *S. planiculmis* was lower than that of cotton (*p <*0.05; **Figure 10J**).

### Effects of spatial distribution of *S. planiculmis* on yield in cotton

**Figure 11:**
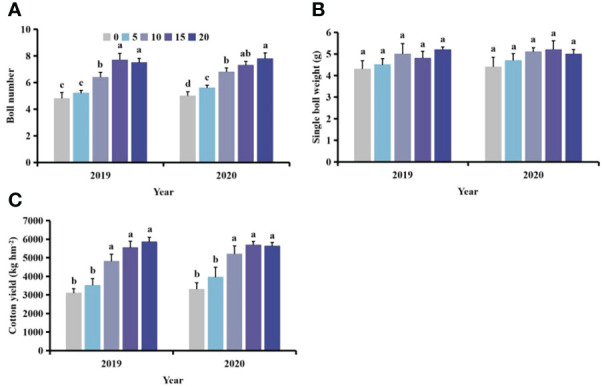
Effects of different spatial distributions of *Scirpus planiculmis* on cotton **(A)** boll number, **(B)** single boll weight and **(C)** cotton yield. The mean value ± standard data were presented in the figure. Different alphabetical letters indicate the significant differences between different distances (*p* <0.05).

With increasing distance between *S. planiculmis* and cotton, the effect of *S. planiculmis* on boll number, single boll weight and yield of cotton decreased (**Figure 11**). Compared with the distance of 20 cm, when the distance was 0 cm, the boll number and yield of cotton decreased by 35.90%-36.00% and 41.33%-47.08%, respectively (*p <*0.05; **Figures 11A, C**). The distance between *S. planiculmis* and cotton had no significant effect on the single boll weight of cotton (*p >*0.05; **Figure 11B**).

### Effects of spraying 0.015 g L^-1^ 75% trifloxysulfuron + 4 g L^-1^ 40% prometryn on physiological indices of cotton and *S. planiculmis*

**Figure 12:**
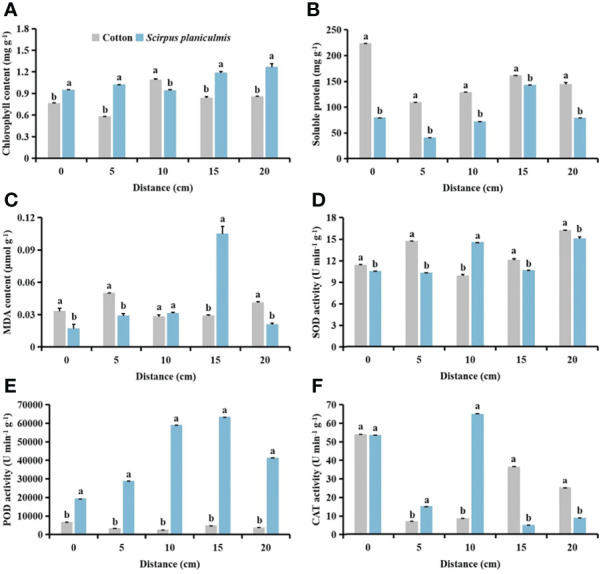
Effects of cotton and *Scirpus planiculmis* on **(A)** chlorophyll, **(B)** soluble protein and **(C)** malondialdehyde (MDA) content, and **(D)** superoxide dismutase (SOD), **(E)** peroxidase (POD) and **(F)** catalase (CAT) activities at different spatial distribution distances of *S. planiculmis* after spraying 0.015 g L^-1^ 75% trifloxysulfuron + 4 g L^-1^ 40% prometryn. The mean value ± standard data were presented in the figure. Different alphabetical letters indicate the significant differences between cotton and *S. planiculmis* (*p* <0.05).

**Figure 13:**
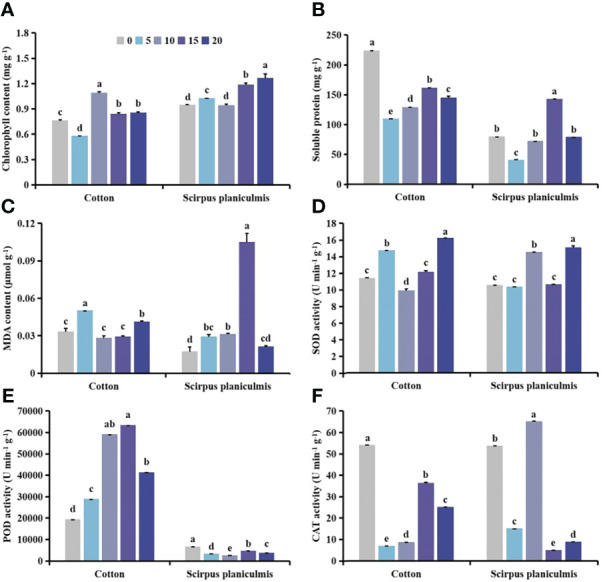
Effects of spraying 0.015 g L^-1^ 75% trifloxysulfuron + 4 g L^-1^ 40% prometryn on **(A)** chlorophyll, **(B)** soluble protein and **(C)** malondialdehyde (MDA) content, and **(D)** superoxide dismutase (SOD), **(E)** peroxidase (POD) and **(F)** catalase (CAT) activities in cotton and *Scirpus planiculmis*. The mean value ± standard data were presented in the figure. Different alphabetical letters indicate the significant differences between different distances (*p* <0.05).

After spraying herbicides, when the distance between *S. planiculmis* and cotton was 10 cm, the chlorophyll content of cotton was higher than that of *S. planiculmis*, increased by 13.77% (*p <*0.05; **Figure 12A**). Under different distance conditions, the soluble protein of cotton was higher than that of *S. planiculmis* (*p <*0.05; **Figure 12B**). When the distance was 15 cm, the MDA content of *S. planiculmis* was 72.38% higher than that of cotton (*p <*0.05; **Figure 12C**). SOD activity of *S. planiculmis* was 31.87% higher than that of cotton only at a distance of 10 cm (*p <*0.05; **Figure 12D**). At all distances, POD activity of *S. planiculmis* was higher than that of cotton (*p <*0.05; **Figure 12E**). When the distance was 0 cm, there was no significant difference in CAT activity between cotton and *S. planiculmis* (*p >*0.05), while at 5−10 cm CAT activity of *S. planiculmis* was higher than that of cotton, and at 15−20 cm the CAT activity of *S. planiculmis* was lower than that of cotton (*p <*0.05; **Figure 12F**). With increasing distance between *S. planiculmis* and cotton, the chlorophyll content of *S. planiculmis* increased (**Figure 13A**). Compared with 0 cm, the soluble protein content of cotton was decreased significantly at 5−20 cm (*p <*0.05; **Figure 13B**). As the distance between *S. planiculmis* and cotton increased, the MDA content of *S. planiculmis* at 5−20 cm was higher than that at 0 cm (*p <*0.05; **Figure 13C**). When the distance was 20 cm, SOD activity of cotton and *S. planiculmis* was highest, at 16.19, and 15.01 U min^−1^ g^−1^, respectively (**Figure 13D**). Compared with 0 cm, the POD activity of cotton was significantly increased at 5−20 cm, while the POD activity of *S. planiculmis* was significantly decreased (*p <*0.05; **Figure 13E**). Compared with 0 cm, the CAT activity of cotton was decreased significantly at 5−20 cm (*p <*0.05), and when the distance was 10 cm the CAT activity of *S. planiculmis* was highest, at 64.99 U min^−1^ g^−1^ (*p <*0.05; **Figure 13F**).

**Figure 14:**
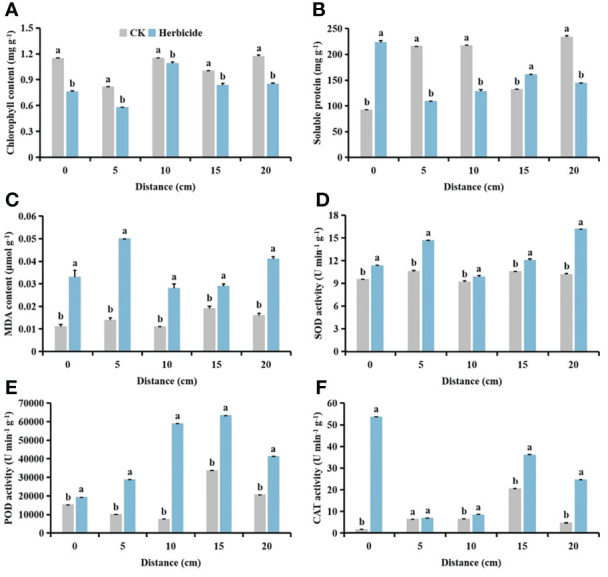
Effects of no herbicide application and spraying 0.015 g L^-1^ 75% trifloxysulfuron + 4 g L^-1^, 40% prometrynon **(A)** chlorophyll, **(B)** soluble protein and **(C)** malondialdehyde (MDA) content, and **(D)** superoxide dismutase (SOD), **(E)** peroxidase (POD) and **(F)** catalase (CAT) activities in cotton at different spatial distribution distances. CK, no herbicide application: Herbicide, spraying 0.015 g L^-1^ 75% trifloxysulfuron + 4 g L^-1^ 40% prometryn. The mean value ± standard data were presented in the figure. Different alphabetical letters indicate the significant differences between CK and herbicide (*p* <0.05).

**Figure 15:**
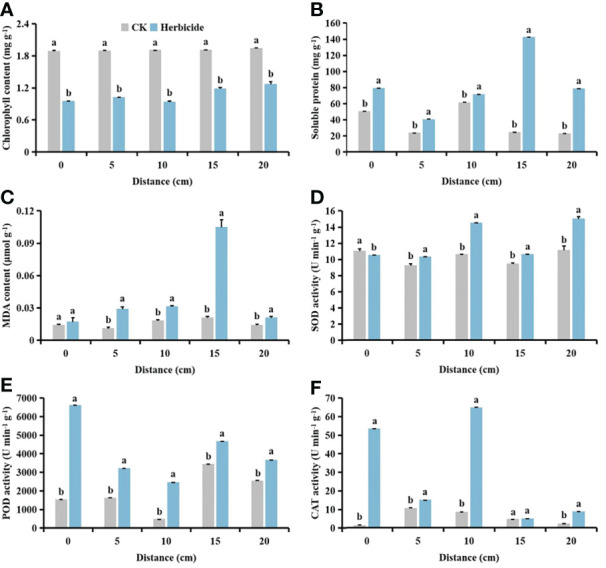
Effects of no herbicide application and spraying 0.015 g L^-1^ 75% trifloxysulfuron + 4 g L^-1^ 40% prometryn on **(A)** chlorophyll, **(B)** soluble protein and **(C)** malondialdehyde (MDA) content, and **(D)** superoxide dismutase (SOD), **(E)** peroxidase (POD) and **(F)** catalase (CAT) activities in *Scirpus planiculmis* at different spatial distribution distances. CK, no herbicide application; Herbicide, spraying 0.015 g L^-1^ 75% trifloxysulfuron + 4 g L^-1^ 40% prometryn. The mean value ± standard data were presented in the figure. Different alphabetical letters indicate the significant differences between CK and herbicide (*p* <0.05).

Compared with no herbicide application, herbicide application decreased the chlorophyll content of cotton and *S. planiculmis* (**Figures 14A** and **15A**), and increased MDA content, and SOD, POD and CAT activities of cotton and *S. planiculmis* (**Figures 14C-F** and **15C-F**). At 0 and 15 cm, application of herbicides increased the soluble protein content of cotton by 58.74% and 17.87%, respectively (*p <*0.05; **Figure 14B**). However, application of herbicides increased the soluble protein content of *S. planiculmis* at all distances (*p <*0.05; **Figure 15B**).

## Discussion

The results of our study showed that with different stress patterns (density, competition period, and spatial distribution) of *S. planiculmis*, protective enzymes were involved in the response of cotton to *S. planiculmis* (**Figures 2**, **6** and **9**). Compared with SOD and POD, cotton increase CAT activity under different stress patterns of *S. planiculmis* (**Figure 2**, **6** and **9**). The protective enzyme activities of cotton and *S. planiculmis* were increased under low dose of herbicides (**Figure 14**, **15**). Due to the lethal effect of herbicides on *S. planiculmis*, this will help cotton to further improve the protective enzyme activity to reduce the stress of *S. planiculmis*.

Cotton is an economically important crop that is very sensitive to weed competition (Wang et al., 2015). In the field, cotton growth and development are easily disrupted by weeds, resulting in dwarf plants, chlorosis, and even a decrease in the number of flowers and bolls, ultimately resulting in reduced cotton yield (Ma et al., 2016). Different weed species exert different interference effects on cotton. Herein, based on the literature, we classified weeds that compete with cotton into three categories according to plant type: low creeping weeds, comprising short plants have a poor shading effect on cotton, including goosegrass (Ma et al., 2015a); weeds with stout stems and lush branches and leaves that have a great influence on the photosynthetic utilization and morphological characterization of cotton, including *Solanum nigrum* (Zhang et al., 2022a), giant ragweed (Barnett and Steckel, 2013), redroot pigweed (Ma et al., 2015b) and velvetleaf (Ma et al., 2016); and intermediate weeds with a form between stout and tall plants and low creeping plants. Although members of this third class are taller, their stems are slender, and their shading effect on cotton is stronger than that of low creeping and weaker than that of stout and tall classes. *S. planiculmis* belongs to this third class (Cai et al., 2020a). Studies have shown that goosegrass in cotton fields does not significantly reduce the plant height of cotton, and at the maximum goosegrass density, the stem diameter and number of sympodial branches of cotton were only reduced by 6% compared with controls (Ma et al., 2015a), indicating that goosegrass had minimal effect on the morphological characteristics of cotton. However, in cotton fields containing *S. nigrum* (Zhang et al., 2022a), giant ragweed (Barnett and Steckel, 2013), redroot pigweed (Ma et al., 2015b) and velvetleaf (Ma et al., 2016), plant height and stem diameter of cotton were significantly affected, and plant height and stem diameter decreased regularly with increasing weed density. For example, for rows containing an extra velvetleaf plant, cotton plant height and stem diameter decreased by 5 cm and 2 mm, respectively (Ma et al., 2016). Similarly, plant height and stem diameter decreased by 2−13 cm and 2 mm, respectively, for each additional redroot pigweed per row (Ma et al., 2015b). This is due to the strong light interception effect of tall and stout weeds on cotton (Scott et al., 2000), which affects morphological characterization. Our results showed that *S. planiculmis* significantly reduced plant height and stem diameter of cotton only under certain density conditions (≥20 plants m^-2^) at budding, and flowering and boll stages (**Figure 2**). This indicates that under low density conditions (<20 plants m^-2^), *S. planiculmis* does not affect the upper morphological characteristics of cotton. In addition, we found that the chlorophyll content of cotton decreased with increasing density of *S. planiculmis* at the flowering and boll stage of cotton, but the chlorophyll content of cotton increased under high density of *S. planiculmis* (80−100 plants m^-2^) at the seedling stage and bud stage of cotton (**Figure 2**). This indicates that the effect of light interception of *S. planiculmis* in the cotton flowering and boll stage was stronger than that in the seedling stage and budding stage, while in the seedling stage and bud stage, a high density of *S. planiculmis* stimulated cotton photosynthesis and increased the chlorophyll content of the crop.

In general, weed damage can affect the entire growth and development cycle of cotton, and long-term persistent stress has an adverse effect on cotton morphogenesis (Charles and Taylor, 2021). Long-term competition by weeds for light and nutrient resources has reduces the ability of cotton to absorb and utilise nutrients, manifesting as slender, short plants (Wang et al., 2015). For example, following continuous damage to cotton by *S. nigrum*, levels of phosphorus, potassium, soluble protein and soluble sugar were reduced, and plant height and stem diameter of cotton were decreased with increasing stress duration (Han, 2021). Similarly, during competition between *S. planiculmis* and cotton, the content of potassium and soluble sugar in cotton decreased with prolongation of the competition period, which was not conducive to the absorption and accumulation of nutrients by cotton, resulting in decreased plant height and stem diameter of cotton (Cai, 2020c). In addition, after continuous competition of sunflower, Japanese millet and mungbean as mimic weeds with cotton, plant height and biomass of cotton were reduced to varying degrees, which had a negative effect on the vegetative growth of cotton (Charles et al., 2019a; Charles et al., 2019b; Charles et al., 2020). In the present study, we found that when *S. planiculmis* was planted in April, there was no significant change in plant height, stem diameter, fresh weight, root length or chlorophyll content of cotton with prolongation of the competition period between *S. planiculmis* and cotton (**Figure 6**). However, when *S. planiculmis* was planted in May and June, plant height, stem diameter, fresh weight, root length and chlorophyll content of cotton decreased with continuation of the competition period (**Figure 6**). This shows that *S. planiculmis* had little effect on the growth and photosynthesis of cotton in the early stages, but it had a great effect on the morphogenesis and photosynthesis of cotton in the middle and later stages. Additionally, we also found that when *S. planiculmis* and cotton competition period for 30 days, the soluble protein content in cotton was lowest, and after 60 days the soluble protein content in cotton gradually returned to normal levels (**Figure 6**). This may be due to long-term competition between cotton and *S. planiculmis*. Cotton enhances the absorption and accumulation of nitrate nitrogen, and converts nitrate nitrogen into soluble protein after assimilation in the body (Marschner, 2012; Cai, 2020c).

Most previous studies ignored the mediating effect of allelopathy on competition between cotton and weeds, but allelopathy of weeds is an objective inducing factor that affects the growth and development of cotton (Wang et al., 2022). For example, extracts of *Medicago sativa*, *Flaveria bidentis*, and *Eclipta prostrata* can inhibit the germination rate and radicle growth of cotton (Yuan et al., 2008; Peng et al., 2011; Yang et al., 2015). Our previous studies have shown that root exudates of *S. planiculmis* have no significant effect on the growth of above-ground parts of cotton, but can significantly inhibit the growth of underground parts, and high concentrations can reduce the content of cytokinin and auxin in cotton (Cai, 2020c). This indicates that allelopathy of *S. planiculmis* affected the growth and development of cotton by inhibiting the synthesis of cytokinin and auxin. It is worth noting that interference of root exudates of *S. planiculmis* on cotton is correlated with concentration, and therefore the strength of allelopathy (Li et al., 2020).

Interference of allelopathy on plants is related to the distance between plants (Li et al., 2022b). In this study, we analysed this effect by changing the distance between *S. planiculmis* and cotton. We found that plant height and stem diameter of cotton did not change significantly under different distance conditions, indicating that allelopathy of *S. planiculmis* did not affect the growth of aboveground parts of cotton, consistent with our indoor research results (Cai, 2020c). However, the difference is that fresh weight and root length of cotton did not change significantly with a difference in distribution distance (**Figure 9**), which may be due to the low density of flat stalks (eight plants) in this study. The allelopathic intensity of *S. planiculmis* was weak at this density, which could not significantly inhibit fresh weight and root length of cotton, while *S. planiculmis* at higher density (≥20 plants/m^2^) caused significant inhibition. This also indicates that allelochemicals of *S. planiculmis* can inhibit growth of the underground parts of cotton at high concentrations (Cai, 2020c).

Under allelochemical stress, receptor plants can alleviate damage caused by reactive oxygen species (ROS) by altering enzyme activity *via* a self-protective mechanism (Li et al., 2022a). Recent studies have shown that the allelopathic effect of *Artemisia argyi* water extract (AAWE) can lead to membrane system rupture and ROS release in rice, and under low AAWE concentration, an increase in SOD activity can compensate for a decrease in CAT and POD activities, which ensures that ROS levels remain relatively stable. However, with increasing AAWE concentration, POD and CAT activities continued to decrease, and MDA content increased, eventually leading to ROS imbalance (Li et al., 2022a). Our study showed that under different densities of *S. planiculmis*, SOD activity of cotton was decreased at each period, while CAT activity was increased, and POD activity was increased in the budding stage and the flowering and boll stage, whereas MDA content was decreased significantly in the flowering and boll stage (**Figure 2**). This shows that POD and CAT are protective enzymes that play a major role in cotton in response to the density effect of *S. planiculmis*. Only CAT activity showed an increasing trend with prolongation of competition period (**Figure 6**). This indicates that CAT in cotton plays a continuous role in resisting *S. planiculmis* under long-term competition. Regarding the distribution of *S. planiculmis*, only CAT activity was increased at different distance ranges (**Figure 9**). Therefore, we believe that an increase in CAT activity plays a key role in the responses of cotton to *S. planiculmis* stress.

We further explored the effects of herbicide application on physiological metabolism of *S. planiculmis* and cotton, and found that protective enzyme activities of both species were improved after herbicide application (**Figure 14** and **15**). The herbicide used in this study was selective and had a good control effect on *S. planiculmis* (Peng et al., 2012; Tian and Yu, 2020). Although the protective enzyme activity of *S. planiculmis* increased after application of low doses of herbicide, this is a normal response of weeds to herbicides (Peng et al., 2013a; Peng et al., 2013b). However, herbicides do not kill cotton; rather, they only stimulate protective enzyme activities (Peng et al., 2013a; Peng et al., 2013b). This indicates that during interaction between cotton and *S. planiculmis*, herbicides may gradually weaken the stress advantage of *S. planiculmis*, enhance the self-protection ability of cotton, and improve the resistance of cotton to *S. planiculmis*. Therefore, we believe that when controlling *S. planiculmis*, application of low-dose selective herbicides may help cotton to resist stress caused by weeds. Our previous studies showed that application of low-dose insecticides, fungicides and plant growth regulators can increase protective enzyme activities of cotton, and help resist pest-associated stress (Zhang et al., 2022c; Zhang et al., 2022d; Zhang et al., 2022e). Clearly, a low-dose pesticide can stimulate the defence response of cotton. On the one hand, pesticides can directly act on pests, and on the other hand, they can enhance the defence ability of cotton against pests, and resistance to pests is promoted through this dual role. This knowledge helps to explain pest resistance, and supports the sustainable development of agricultural production management.

It cannot be ignored that intraspecific competition of weeds is always accompanied by interactions between weeds and cotton (Wang et al., 2015). In previous reports, it was confirmed that during competition between cotton and giant ragweed (Barnett and Steckel, 2013), goosegrass (Ma et al., 2015a), redroot pigweed (Ma et al., 2015b) or velvetleaf (Ma et al., 2016), intraspecific competition of weeds led to a decrease in the biomass of individual plants. Specifically, the stem diameter of redroot pigweed and velvetleaf decreased with increasing density, while plant height increased (Ma et al., 2015b; Ma et al., 2016). We found that with increasing population density, plant height of *S. planiculmis* tended to increase, while stem diameter showed a downward trend, and fresh weight and root length first increased then decreased (**Figure 1**). Moreover, *S. planiculmis* responds to intraspecific competition by regulating photosynthesis, soluble protein synthesis, and protective enzyme activities, *via* a self-regulatory strategy characteristic of weed populations (Li et al., 2022b).

Weeds ultimately impair cotton yield by altering cotton plant morphology and nutrient metabolism (Wang et al., 2015). In this study, with increasing *S. planiculmis* density, cotton boll number, single boll weight and yield were diminished (**Figure 4**). In addition, continuous damage by *S. planiculmis* also led to a decrease in boll number, single boll weight and yield (**Figure 8**). However, with increasing distance between *S. planiculmis* and cotton, boll number, single boll weight and yield were less affected (**Figure 11**). Using equation fitting, we previously determined that the control threshold of *S. planiculmis* in cotton fields was 20 plants m^-2^, and the duration of damage should be less than 30 days (Cai et al., 2020a, 2021). Therefore, we suggest that *S. planiculmis* should be controlled in the field to avoid significant loss of cotton yield.

Moreover, temperature and rainfall are considered to be important climatic factors affecting plant growth (Khan et al., 2022; Kharivha et al., 2022). In order to adapt to abiotic stresses such as drought, high temperature and low temperature, plants also reduce the impact of climatic conditions on themselves by regulating defense responses (Akpinar and Cansev, 2022; Vital et al., 2022). Obviously, when studying the stress of weeds on cotton in the field, extreme climate may have an impact on cotton response to weed stress. Therefore, climate factors need to be included in this consideration. We documented changes in temperature and precipitation during the cotton growing stage (seedling to boll opening) from 2019 to 2020 (**Figure S1**). These climatic data reflect the characteristics of large temperature difference between day and night and low rainfall in the test area, which belongs to the typical temperate continental arid climate. Temperature difference led to the acceleration of plant growth and the occurrence of phenological events (Talukder et al., 2014; Lymperopoulos et al., 2018). To adapt to the environment with large temperature difference, cotton improves the adaptability to temperature difference by regulating photosynthesis, respiration and physiological metabolism (Wang et al., 2016, 2018). In order to reduce the effect of temperature difference on cotton, we chose plastic film mulching to maintain soil temperature. Especially in low temperature conditions, the film has played a role in raising soil temperature, which can reduce the temperature difference on cotton. Studies have shown that with the strengthening of drought stress, cotton plant height, stem diameter and other morphological indicators decreased, and by regulating photosynthesis and protective enzyme activity in a sustained manner to respond strongly to drought (Tian et al., 2017). In this study, we made up for the lack of rainfall and avoided the occurrence of drought stress by drip irrigation. In addition, the film played a good role in moisture conservation. Therefore, we think that the effects of climatic factors on cotton growth and physiology can be reduced by applying reasonable cultivation and management measures.

In summary, using *S. planiculmis* and cotton as an example, we analysed the stress effect of *S. planiculmis* on cotton under different densities, competition periods and distribution conditions, from the perspective of morphogenesis, physiological metabolism and yield, and revealed the defence pathway of cotton in response to *S. planiculmis* that involves regulation of protective enzyme activities. We concluded that CAT plays a key role in protecting enzymes involved in defence responses. On this basis, we further explored the effect of low-dose herbicide on the interaction between cotton and *S. planiculmis*. The results showed that low-dose herbicide could help cotton resist stress caused by *S. planiculmis* by elevating the activities of protective enzymes in cotton. Therefore, we believed that when attempting to control *S. planiculmis*, low doses of herbicide may protect cotton and control weeds. This strategy is also applicable to the control of other pests. In the future, we will further explore the response strategy of cotton to *S. planiculmis* from the perspective of gene regulation, and thereby obtain a deeper explanation for the stress mechanism of cotton in response to *S. planiculmis*.

## Data availability statement

The raw data of the presented results of this study are available on request to the corresponding author.

## Author contributions

QZ wrote the manuscript; JW designed the study; JP conducted experiments; QZ and JP analyzed data. All authors contributed to the article and approved the submitted version.

## Funding

This research was supported by the National Natural Science Foundation of China (Grant No. 31260435, 31660519), and the Xinjiang Corps Major Science and Technology Project (Grant No. 2018AA06-02).

## Acknowledgments

We thank reviewers and editors for valuable comments and suggestions on the manuscript.

## Conflict of interest

The authors declare that the research was conducted in the absence of any commercial or financial relationships that could be construed as a potential conflict of interest.

## Publisher’s note

All claims expressed in this article are solely those of the authors and do not necessarily represent those of their affiliated organizations, or those of the publisher, the editors and the reviewers. Any product that may be evaluated in this article, or claim that may be made by its manufacturer, is not guaranteed or endorsed by the publisher.
