# Supplementary figures and images for: Protective enzyme activity regulation in cotton (Gossypium hirsutum L.) in response to Scirpus planiculmis stress
Source: Front Plant Sci. 2022 Nov 28;13:1068419. doi: 10.3389/fpls.2022.1068419 (PMC9742448; doi:10.3389/fpls.2022.1068419)

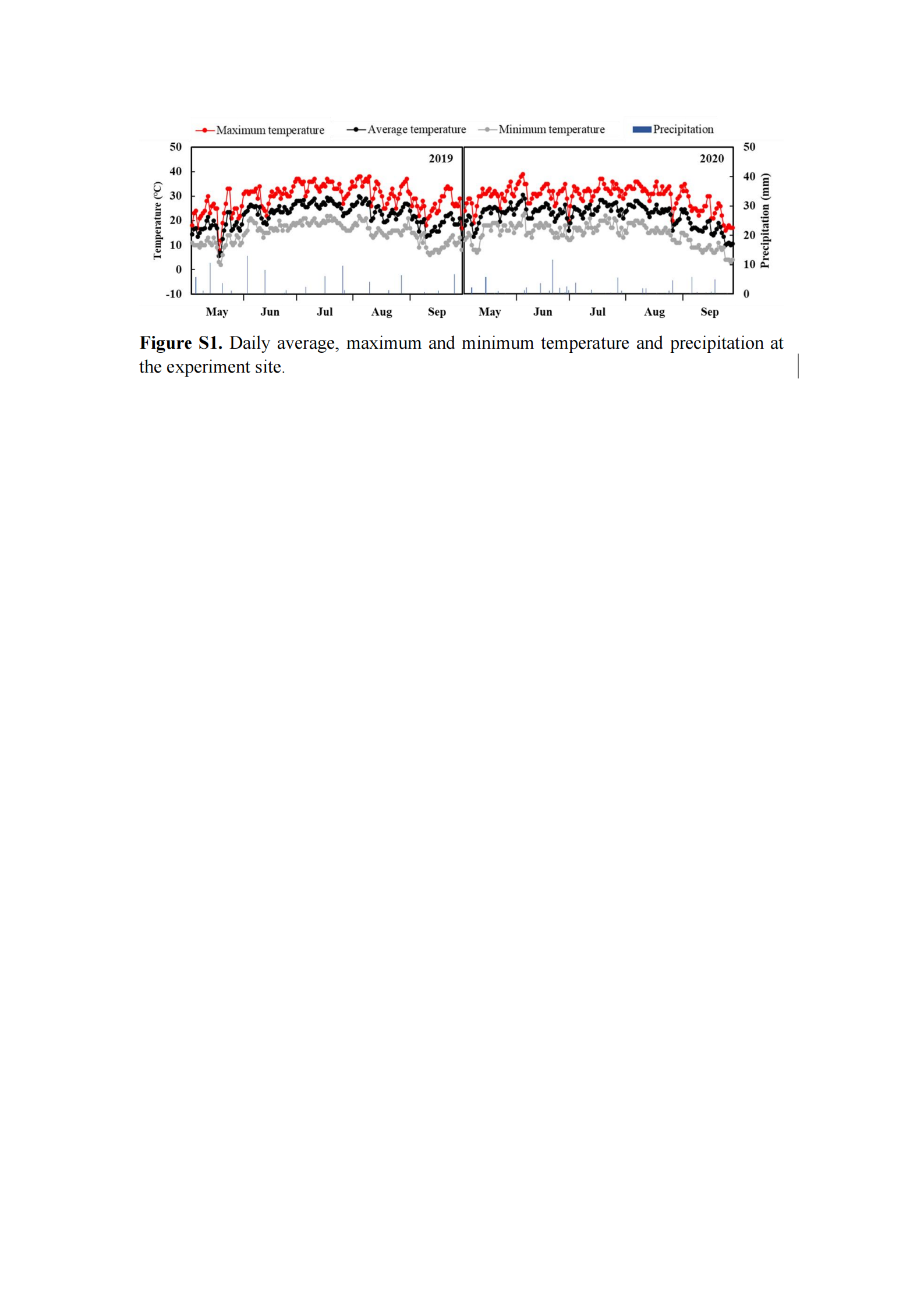

Supplement: Supplementary file 1 [file Image_1.tif]
